# Acoustic wave science realized by metamaterials

**DOI:** 10.1186/s40580-017-0097-y

**Published:** 2017-02-07

**Authors:** Dongwoo Lee, Duc Minh Nguyen, Junsuk Rho

**Affiliations:** 1 Department of Naval Architecture and Ocean Engineering, Mokpo National Maritime University, Mokpo, 58628 Republic of Korea; 2 Department of Mechanical Engineering, Pohang University of Science and Technology (POSTECH), Pohang, 37673 Republic of Korea; 3 Department of Chemical Engineering, Pohang University of Science and Technology (POSTECH), Pohang, 37673 Republic of Korea

**Keywords:** Metamaterials, Sub-wavelength, Cloaking, Sub-diffraction imaging, Gradient index

## Abstract

Artificially structured materials with unit cells at sub-wavelength scale, known as metamaterials, have been widely used to precisely control and manipulate waves thanks to their unconventional properties which cannot be found in nature. In fact, the field of acoustic metamaterials has been much developed over the past 15 years and still keeps developing. Here, we present a topical review of metamaterials in acoustic wave science. Particular attention is given to fundamental principles of acoustic metamaterials for realizing the extraordinary acoustic properties such as negative, near-zero and approaching-infinity parameters. Realization of acoustic cloaking phenomenon which is invisible from incident sound waves is also introduced by various approaches. Finally, acoustic lenses are discussed not only for sub-diffraction imaging but also for applications based on gradient index (GRIN) lens.

## Introduction

Metamaterials made of periodic or random artificial structures, defined as “meta-atoms” with size that is larger than the conventional atom and much smaller than the radiated wavelength, are used for deeply control and manipulation of waves. Since the properties of the metamaterials are governed by the meta-atom structures rather than their base materials, by careful designing and engineering the parameters of the meta-atom structures such as shape, geometry, size or orientation, fascinating functionalities beyond the capability of conventional materials can be realized. The concept of metamaterials was first proposed by Veselago [1] in 1968 for electromagnetic waves, but it needed to wait for around 30 years for the next step when Pendry reported artificial designs with effectively negative permeability and permittivity in 1999 [2, 3]. Metamaterials were then experimentally demonstrated by Smith and Shelby [4, 5] for negative refractive index structures and have since been a subject of numerous studies in a wide variety of wave-matter interaction, including not only photonics but also acoustic wave science.

Acoustic wave science studies the propagation of matter oscillation through an elastic medium such as air or water and therefore explains energy transfer through the medium. While the movement of oscillating materials is limited through its equilibrium position, vibrational waves can propagate in a long distance and can be reflected, refracted, attenuated or, more generally, manipulated by the medium. According to the oscillation frequency, acoustic waves have been classified to different fields that cover the audio, ultrasonic and infrasonic frequency range, or seismic waves at much larger scale which are waves of energy travelling through the Earth’s layer.

The advent of fabrication technology [6–8] together with development of simulation techniques such as finite element method (FEM) and finite difference time domain method (FDTD) have led to a revolution of metamaterials in controlling and manipulating acoustic waves in new ways not previously imagined [9, 10]. For instance, in acoustics, it is now possible to design acoustic lenses for sub-diffraction imaging [9–12] or design acoustic cloaking which is able to make an object acoustically invisible by bending the waves [13–16]. Also, an assembly of rubber-coated spheres into a bulk metamaterial can exhibit locally designed resonant structures [17].

Our objective here is to present a unified discussion of the advances of metamaterials in acoustic wave science. The review is organized as follows. We focus on the acoustic metamaterials in Sect. 2 with discussions in theory about acoustic parameters such as mass density and bulk modulus. The section is then followed by our review of metamaterial designs for controlling these two parameters to achieve unusual negative or near-zero values that cannot be found in nature. As a next part, acoustic cloaking is discussed in detail with different approaches. Lastly, superlens and hyperlens for sub-diffraction imaging are organized then, Luneburg and Eaton lens which are based on the concept of (GRIN) lens are introduced.

## Acoustic metamaterials

Propagation of acoustic waves including sound waves in the audio frequency range is controlled by the mass density and the bulk modulus of a material through acoustic wave equation

1 $$\nabla^{2} P - \frac{\rho }{B}\frac{{\partial^{2} P}}{{\partial t^{2} }} = 0$$

where *P* is the pressure and $\rho ,B$ are the mass density and bulk modulus of materials, respectively. Physically, the mass density is defined as mass per unit volume and the bulk modulus reflects the medium’s resistance to external uniform compression. These two parameters are analogous to the electromagnetic parameters, permittivity $\varepsilon$ and permeability $\mu ,$ as can be seen in the following expression of the refractive index *n* and the impedance *Z*.

2 $$n = \sqrt {\frac{\rho }{B}} \, ({\text{acoustics}})\quad n = \sqrt {\varepsilon \mu } \, ({\text{electromagnetism}})$$

3 $$Z = \sqrt {\rho B} \, ({\text{acoustics}})\quad Z = \sqrt {\mu /\varepsilon } \, ({\text{electromagnetism}})$$

**Fig. 1:**
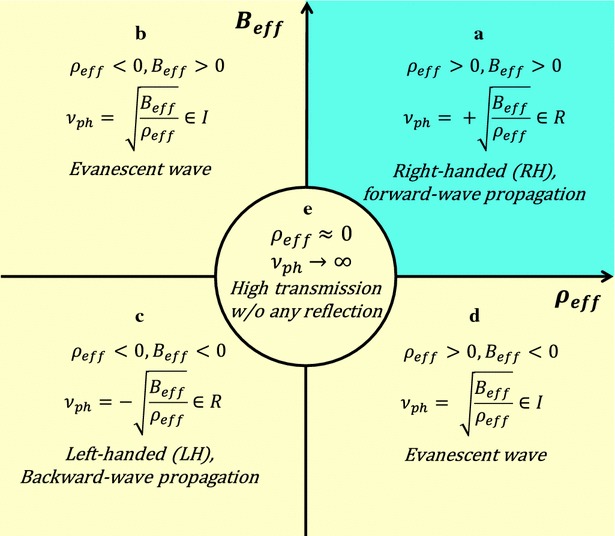
Effective mass density and bulk modulus diagram. *v*_*ph*_ denotes the phase velocity. *R* and *I* indicate purely real and imaginary parts of phase velocity, respectively. Normal materials in nature belong to **a** with double positive parameters. Metamaterials can be classified as negative mass density (**b**), double negative parameters (**c**), negative bulk modulus (**d**) and near-zero mass density (**e**)

The mass density and the bulk modulus are always positive in conventional media and hard to modify because the material properties are directly associated with the chemical composition and bonding structures of the constituted atoms. However, a variety of effective acoustic parameters including negative values which never existed in nature can be obtained by metamaterials whose properties are mainly governed by the meta-atom structures that behaves like a continuous material in the bulk. According to the sign of the mass density and the bulk modulus, acoustic metamaterials can be classified to negative mass density, negative bulk modulus, double negative parameters, near-zero and approaching-infinity mass denstiy as shown in Fig. 1. These types of acoustic metamaterials together with each corresponding applications will be directly in the next sub-sections.

### Negative mass density

**Fig. 2:**
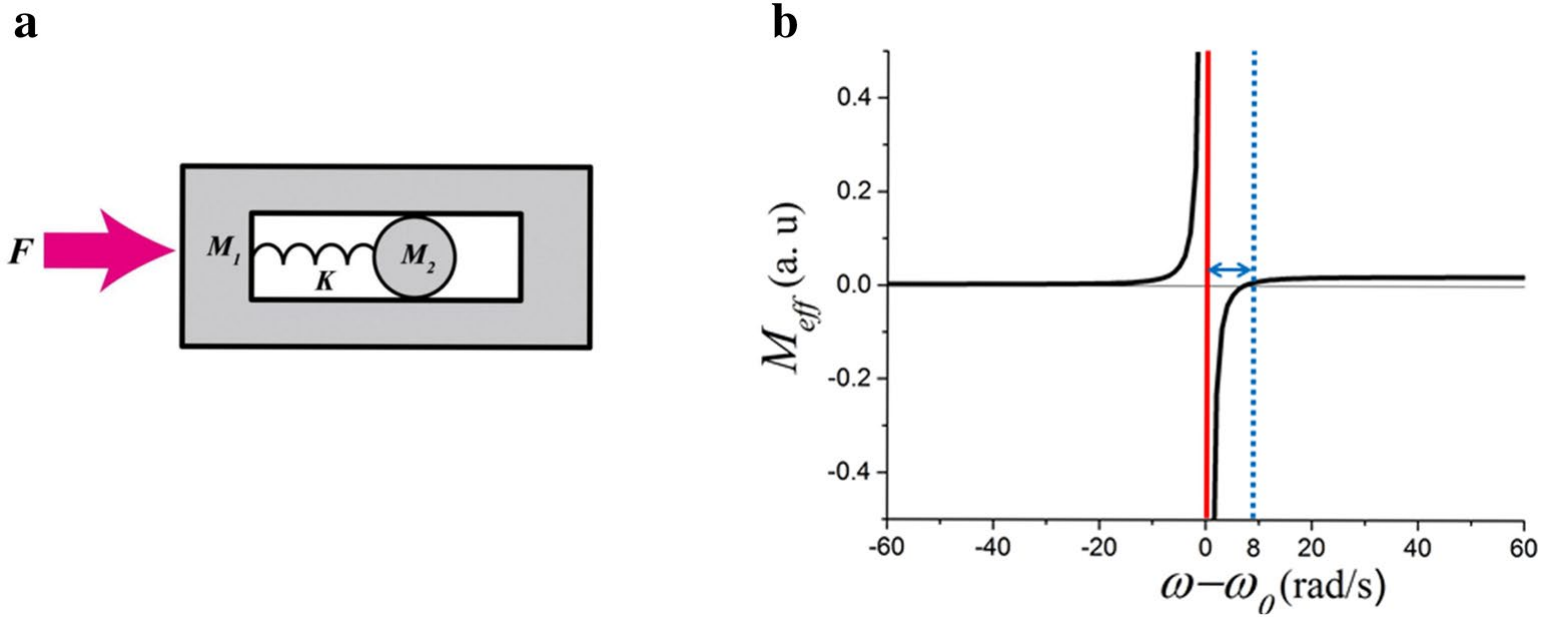
**a** Simple mass-spring system. **b** Effective inertial response as a function of angular frequency obtained from Eq. (5) where *M*_1_, $\omega_{0}$ and *K* are set to 0.002, 0 and 1, respectively. **a** is adapted from [19]

When an atom is deviated from the equilibrium state, it will be pulled back to the balance position by a central force explained by Newton’s second law $F = m\ddot{x}.$ Although the mass of an atom must be always positive, negative effective mass density can be achieved in a periodic structure comprising of artificial meta-atoms near its resonant frequency. The physical nature of effective mass density was theoretically explained by Milton and Willis [18] through a mass-spring system. A simple mass-spring system consisting of mass *M*_2_ positioning inside the cavity of mass *M*_1_ and coupling with the mass *M*_1_ through a spring of strength *K* is shown in Fig. 2a. If we assume that the masses vibrate without friction under an external force $F(\omega )$ with an angular frequency $\omega ,$ moving equations given by Newton’s second law are described as

4 $$\left\{ \begin{aligned} M_{1} \ddot{x}_{1} - K(x_{2} - x_{1} ) = F \hfill \\ M_{2} \ddot{x}_{2} - K(x_{2} - x_{1} ) = 0 \hfill \\ \end{aligned} \right.$$

where $x_{1} ,\,x_{2}$ are displacements of *M*_1_, *M*_2_, respectively and $\omega_{0} = \sqrt {K/M_{2} }$ is the local resonance frequency. By assuming *x*_1_, *x*_2_ and *F* are time-variant values and solving these differential equations for the external force $F(\omega ),$ we have

5 $$F = \left( {M_{1} + \frac{K}{{\omega_{0}^{2} - \omega^{2} }}} \right)\ddot{x}_{1}$$

The above equation indicates that, in the view of external force, the two-object system *M*_1_–*M*_2_ can be considered as a homogenous one-object system with the resonant frequency $\omega_{0}$ and an effective mass is

6 $$M_{eff} = M_{1} + \frac{K}{{\omega_{0}^{2} - \omega^{2} }}$$

One can deduce from this equation that the effective mass *M*_*eff*_ can be negative if the external force oscillates near the resonant frequency of the system, particularly, in the range $\omega_{0} < \omega < \sqrt {K/M_{1} + \omega_{0}^{2} }$ as can be seen in Fig. 2b. Finally, we have an effective mass density $\rho_{eff}$ by dividing *M*_*eff*_ by the system volume. The term “effective” will be often omitted when describing effective mass density and effective bulk modulus in this review.

**Fig. 3:**
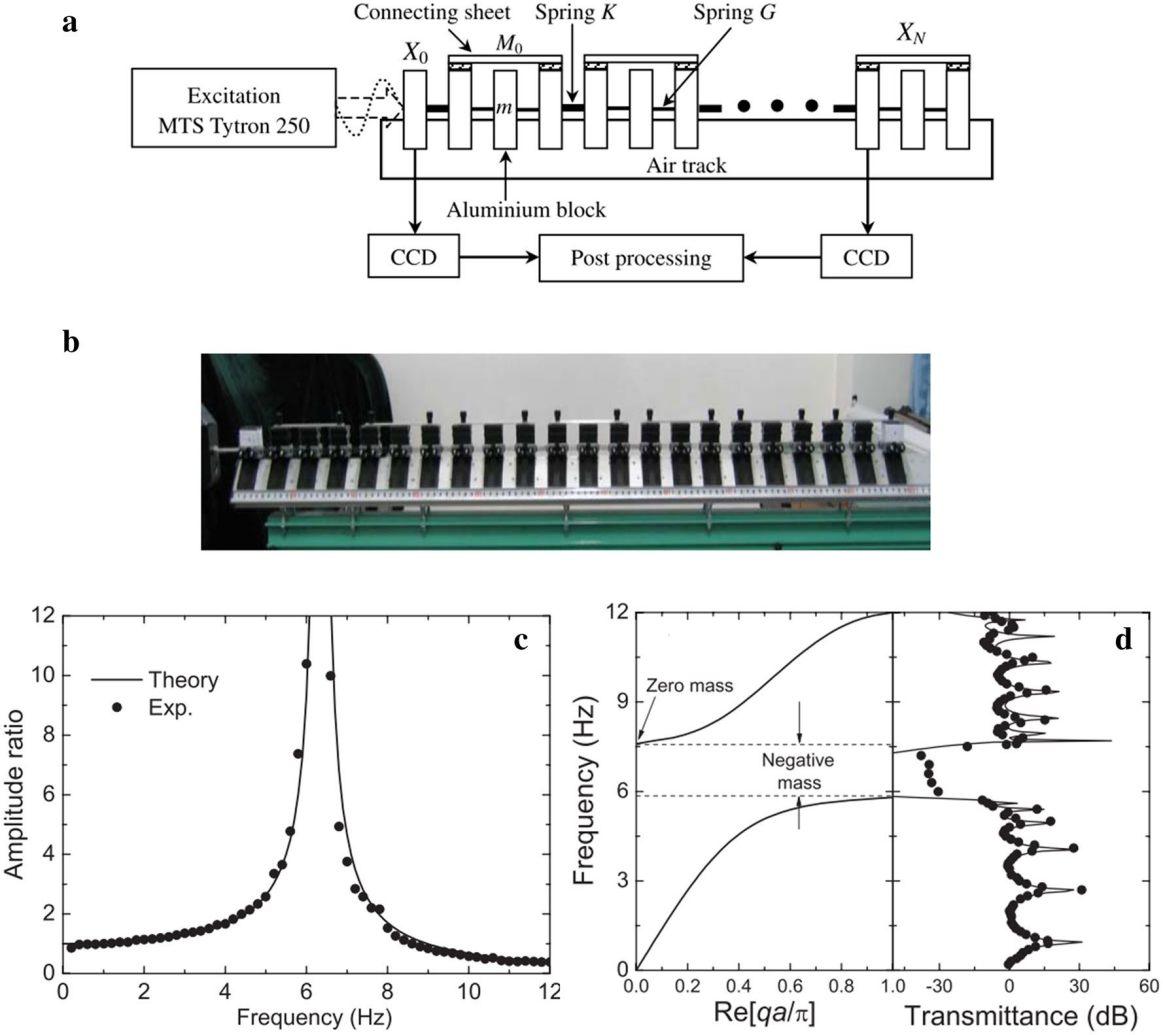
Experiment of 1D spring-mass system. **a** Setup scheme. **b** Actual picture of the experimental setup. **c** Ratio between displacement amplitudes of masses *m* and *M*_0_ for a single unit cell. **d** *Left*-hand side shows the dispersion relation predicted (*q* is the Bloch wave-vector and *a* is the lattice constant). *Right*-hand side shows the transmittance for the whole system and the negative transmittance indicates a negative mass density. **a**–**d** are adapted from [20]

The periodic 1D mass-spring system was experimentally visualized by Yao et al. [20] and recently summarized in [21, 22]. Figure 3a represents the experimental setup which consists of seven unit cells, air track and the harmonic oscillation generator MTS Tytron 250. More particularly, each unit cell is composed of three blocks of length 30 mm, in which first and last blocks are constrained to an aluminum sheet on the top, while the middle block can move freely. The three blocks are attached each other by two soft springs *G* and unit cells are connected to each other by a spring *K*. The dynamic system is finally excited with a harmonic external force with non-friction condition by the MTS Tytron 250 and air track. Actual picture of the experimental setup is shown in Fig. 3b and corresponding measurement results for a single unit cell (Fig. 3c) indicates a strong resonance near 6 Hz. Harmonic movement of the whole system with seven unit cells is also measured as shown in Fig. 3d. As a result, negative mass density was found with a ban-gap near the resonant frequency from about 6 to 7.6 Hz and transmittance defined as the amplitude ratio of *X*_*N*_/*X*_0_ was obtained as around −30 dB.

Generally, negative mass density in acoustic metamaterials can be realized by replacing the mass-spring system to any kind of system having constitutive compositions corresponding to a mass and a spring. For example, a membrane system having a unit cell made up of a rigid grid is reported in [23] where the rigid grid and membrane play the role of the mass and spring, respectively. Such a membrane system with negative mass density has been applied to realize sound absorbers [24–29].

### Negative bulk modulus

**Fig. 4:**
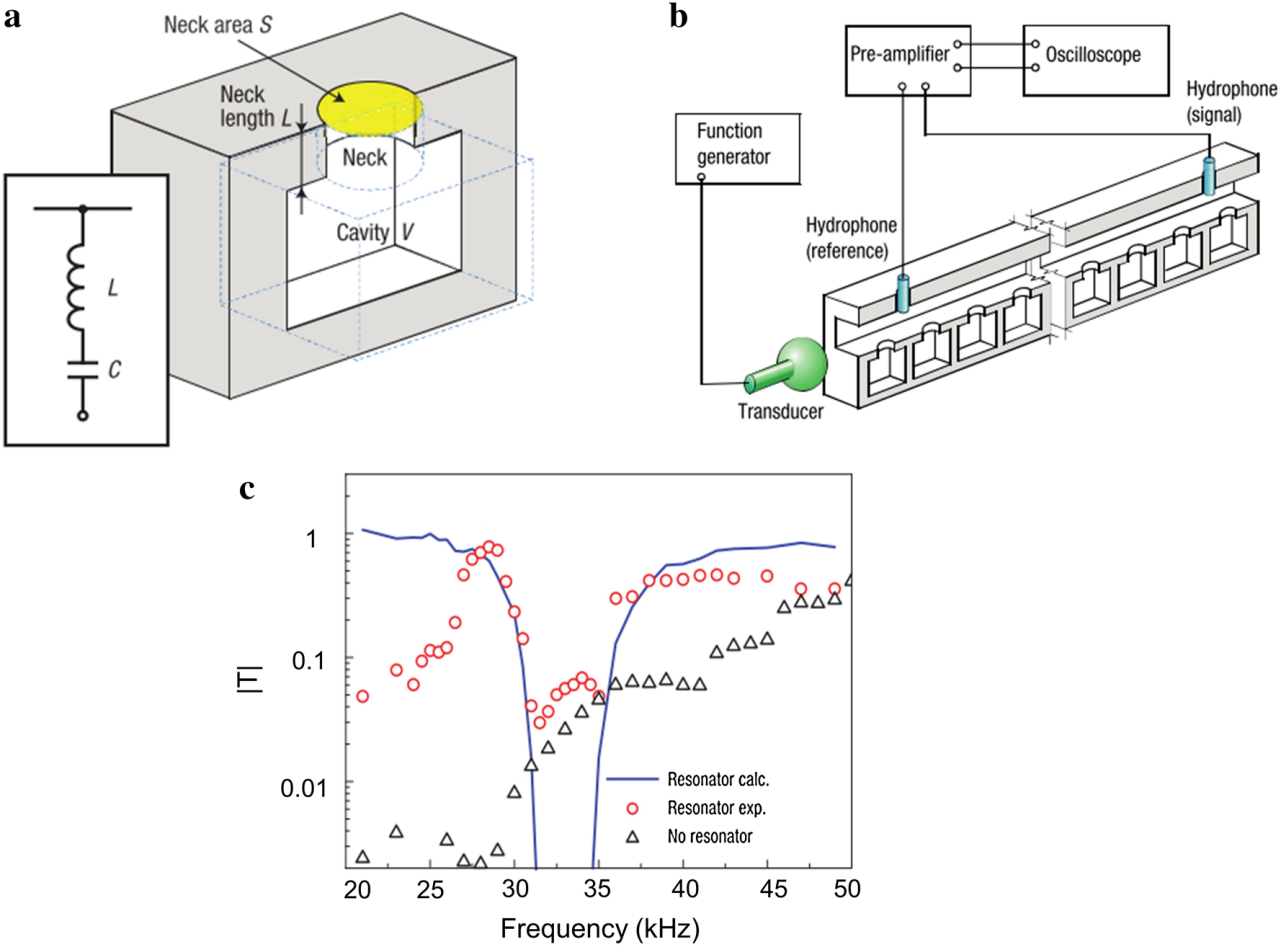
**a** Simple Helmholtz system. **b** Illustration of the setup with connected Helmholtz resonators in series filling water in the cavity. **c** Measured and calculated transmission. Band-gap for attenuation was observed from 31 to 35 kHz. **a**–**c** are adapted from [30]

The property of bulk modulus indicates how the material resists to an external pressure, which is given by

7 $$\Delta P = - B\frac{\Delta V}{V}$$

where Δ*P*, Δ*V*/*V* and *B* denote the pressure change, volume strain and bulk modulus, respectively. Like negative mass density, negative bulk modulus can also be realized by introducing the definition of negative effective bulk modulus in acoustic metamaterials. A simple example of the negative bulk modulus system is a Helmholtz resonator that is basically made up of a large cavity and a narrow neck as shown in Fig. 4a. The effective bulk modulus is expressed by Fang et al. [30]

8 $$B_{eff}^{ - 1} = B_{0}^{ - 1} \left[ {1 - \frac{{F\omega_{0}^{2} }}{{\omega^{2} - \omega_{0}^{2} + i\varGamma \omega }}} \right]$$

where *F* is the geometrical factor, $\omega_{0}$ is the resonant angular frequency and $\varGamma$ is the dissipation loss in the resonating Helmholtz elements. Once again, we can see from the above equation that effective bulk modulus can reach a negative value when the external force oscillates near the resonant frequency. This phenomenon relates to the fact that the cavity is expanded due to an outward restoring force in near the resonant frequency, which indicates the negative bulk modulus. Whereas, being shrunk of the cavity due to an external compressive force indicates the positive bulk modulus in a conventional case. The incoming sound through the neck and the cavity inside are analogous to a mass and a spring, respectively.

The negative bulk modulus system was experimentally demonstrated by Fang et al. and Lee et al. [30, 31]. Fang’s group conducted an underwater ultrasonic transmission experiment composed of Helmholtz resonators in series. The experimental setup shown in Fig. 4b consists of a transducer for underwater sound source and two hydrophones for detection of the signals. Extremely low transmission was observed as visualized in Fig. 4c, indicating that the propagation wave was transformed to evanescent form due to the negative bulk modulus of the metamaterials. Moreover, a formation of negative phase velocity was also confirmed in this experiment due to the loss of friction in the system. Other related works for different types of Helmholtz resonators can be found in [32–35].

**Fig. 5:**
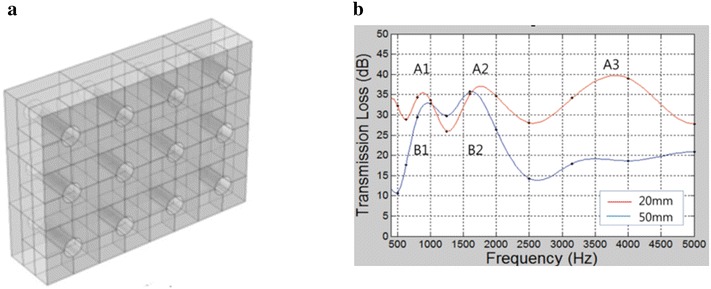
**a** Scheme of the air transparent soundproof window. It consists of transformed resonators and air filters. The diameters of air holes are 20 and 50 mm, respectively. The dimension of each cell is made of 150 mm × 150 mm × 40 mm and a thickness of 5 mm of transparent acrylic. It is arranged an array of 3 × 4 × 3 cells in parallel and series positions. **b** Transmission loss of the air transparent soundproof window. The *red* and *blue lines* denote 20 and 50 mm windows, respectively. The transmission loss is about 30–35 dB in the range of 400–5000 Hz with 20 mm window and about 20–35 dB in the range of 700–2200 Hz with 50 mm window. **a**, **b** are adapted from [36]

An example of application technique for negative bulk modulus is reported by Kim [36] for an air transparent sound proof window. Figure 5a represents the device scheme and the corresponding measurement results are shown in Fig. 5b. One can see that the amplitudes of sound waves are exponentially reduced by demonstrating a successful realization of negative bulk modulus. Moreover, wind pressure can be dropped because the air flow is led to the air holes smoothly. Such a device is useful for the place against huge wind pressure environments such as in hurricane and typhoon.

### Double negative parameters

We have explained in previous sections that either effective mass density or effective bulk modulus of acoustic parameters can be negative near resonant frequency of a periodic artificial structure and then a fully opaque acoustic material is possible. However, an inverse effect in which sound wave energy propagates instead of attenuation will occur when both these two parameters are negative simultaneously.

**Fig. 6:**
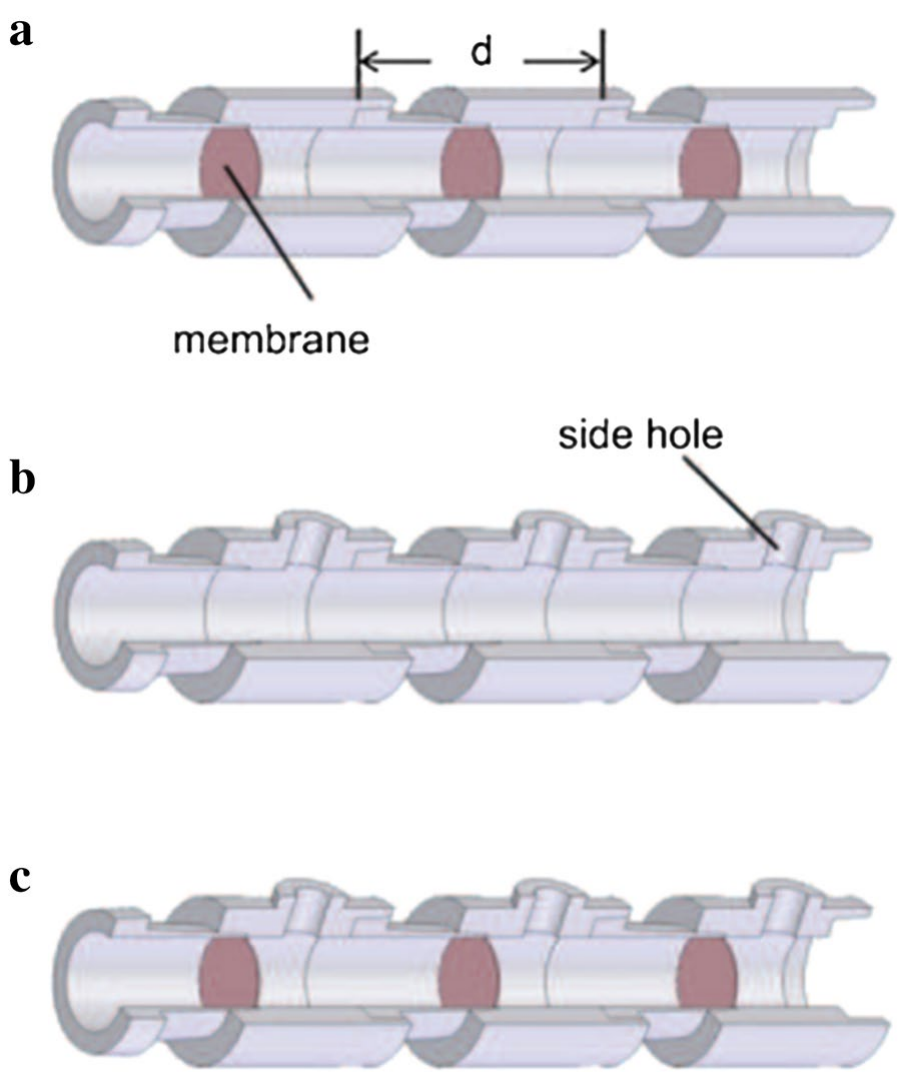
**a** Membrane-type pipe. Membranes are inserted in each lattice constant *d* which is 7 cm, resulting in the negative mass density. **b** Helmholtz-type pipe. Side holes are inserted in series with lattice constant *d*, resulting in the negative bulk modulus. **c** Both membranes and side holes are inserted together, resulting in the negative phase velocity. **a**–**c** are adapted from [41]

In a mechanical system, a dipole resonance is related to the effective mass density because the resonance vibrates along a certain direction, resulting in the inertial response and oscillating like a spring-mass system [17, 23, 37, 38]. A monopole resonance, however, vibrates in all directions associated with a compressive or expansive motion which functions like the change of volume of Helmholtz resonator and is thus related to the effective bulk modulus [37, 39, 40]. Therefore, to realize double negative parameters scheme in Fig. 1c, two resonance symmetries including dipole and monopole resonances must be exploited. The two resonance types can be obtained using membrane and Helmholtz structures. In this manner, Lee et al. [41] demonstrated double negative parameters system for negative phase velocity by combining Helmholtz-type and membrane-type pipes with periodic side holes and membranes as represented in Fig. 6c. Fok and Zhang [42] also tried to demonstrate double negative parameters using rod-spring and Helmholtz structures, but they pointed out that negative refractive index can still be achieved by designing an acoustic metamaterial with negative bulk modulus and positive mass density due to large material loss.

The above methods are limited to a extremely narrow frequency range and more recent researches have continued to overcome this limitation, leading to novel class of acoustic metamaterials so called “space-coiling metamaterials” having negative refractive index over broad range of frequency [43–48]. This kind of metamaterial is realized by coiling up space with curled channels and no requirements for creating local resonances, and can be constructed easily not only for two dimensions but also for three dimensions. We will go back to this type of metamaterial later in Sect. 2.5. Another method for obtaining metamaterials with negative refractive index is to stack several holey plates forming hyperbolic dispersion with highly anisotropic structure [10, 11]. The hyperbolic acoustic metamaterials will be discussed in more detail in Sect. 2.7.2.

### Near-zero and approaching-infinity mass density

**Fig. 7:**
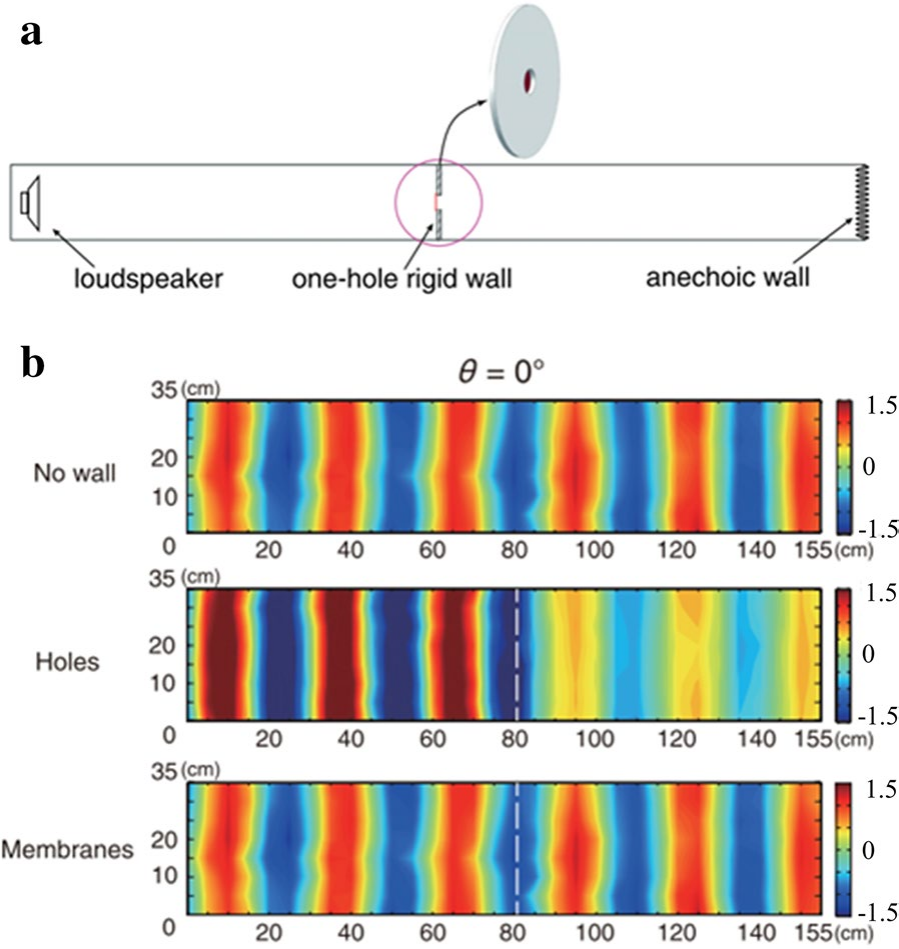
**a** Experimental scheme for near-zero mass density. **b** Instantaneous 2D pressure distributions measured at 1.2 kHz for incidence angle *θ* = 0° are measured for three cases: no wall, presence of holes and presence of membranes. **a**, **b** are adapted from [54]

Another type of acoustic metamaterial is explained in Fig. 1e with near-zero effective mass density. Ideally, this class of metamaterials enables zero refractive index and infinity phase velocity, leading to wave propagation without any reflection and phase change [49–51]. The metamaterials have recently realized by squeezing the sound through ultra-narrow channels [52], embedding a single cylindrical defect which is almost ideal rigid with the sound hard boundary conditions [53] and coiling up space with curled channels [43]. A highlight work related to the near-zero mass density metamaterials is reported in [54]. The metamaterial structure using a thin perforated circular membrane is schemed in Fig. 7a. The setup is composed of a one-hole rigid mounted-circular wall in a circular tube of 2.3 m length and 100 mm inner diameter and a membrane of 17 mm diameter hole at the center. Measurement results of instantaneous 2D pressure distributions at 1.2 kHz for normal incidence presented in Fig. 7b demonstrate a perfect transmission in which both amplitude and phase of the intensity distribution are nearly identical between the case without presence of wall and the case with presence of membranes of the structure.

Another interesting characteristic can be achieved when the effective mass density approaches to infinity. In this case, the impedance in the slab would be very large, leading to large impedance mismatch between the slab and background, and therefore resulting in the nearly total reflection on the interface. This characteristic is demonstrated based on membrane-type acoustic metamaterials and could be exploited in noise control [25, 55].

### Space-coiling metamaterials

**Fig. 8:**
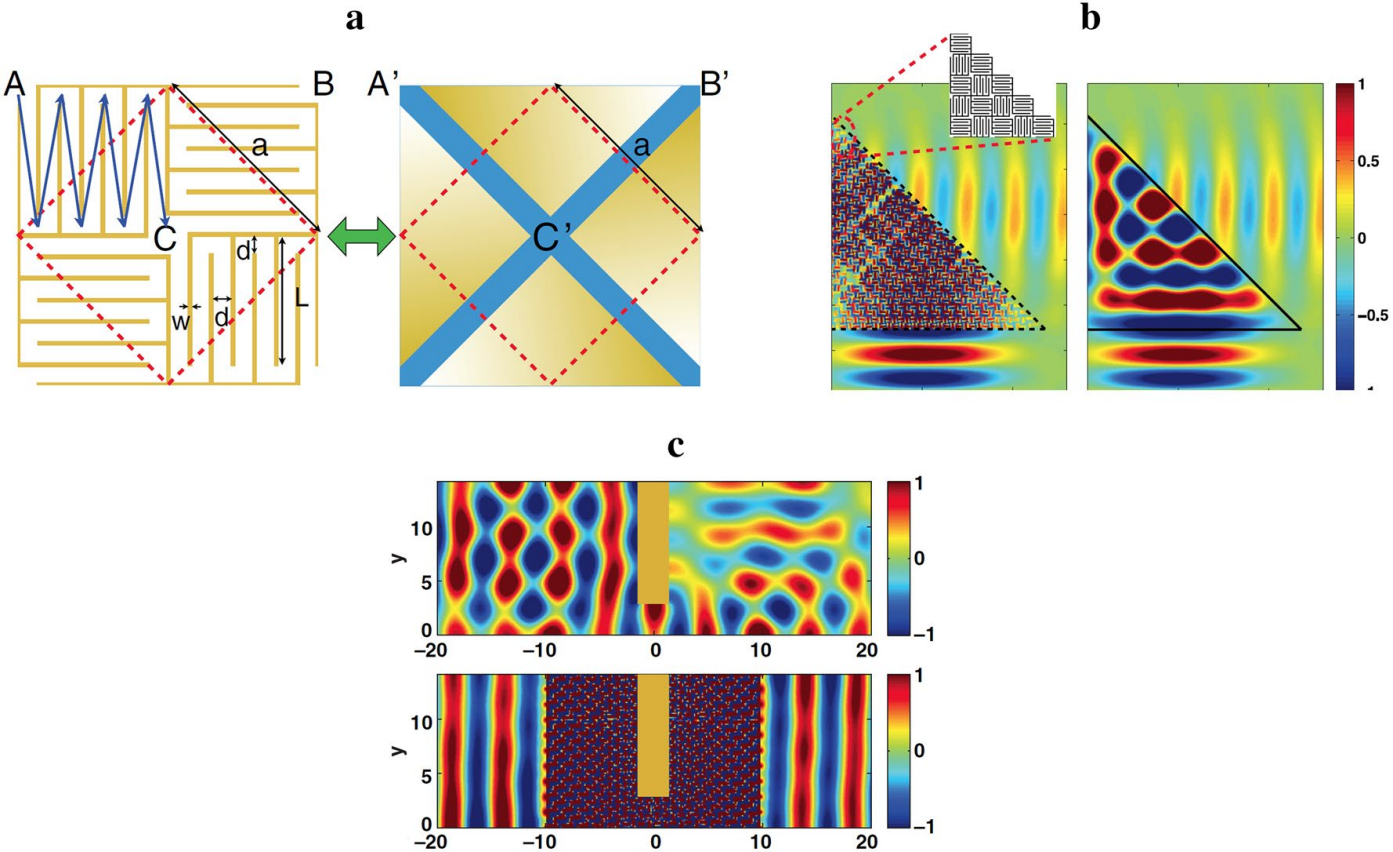
Space-coiling metamaterials. **a** Scheme of a single curled unit (*left*-hand side). It consists of thin plates (length *L*, thickness *d*) arranging in channels of width *d* and lattice constant *a*. The *zigzag arrows* denote a path of waves in the second quadrant inside curled channels. *X*-*shaped blue region* shows a simple view of the path of the waves through the curled channels (*right*-hand side). **b** Pressure field of the space-coiling metamaterials (*left*-hand side) and the effective medium (*right*-hand side) which has same conditions without coiling. It shows both are well matched and the negative refractive index is obtained. **c** Pressure field for the cases of a hard solid plate (*above*) and coiling metamaterials surrounding a hard plate (*below*). High transmission with no reflection by coiling metamaterials is obtained. **a**–**c** are adapted from [43]

Space-coiling metamaterials, known as a subset of double negative parameters in acoustic metamaterials (see Sect. 2.3), have recently drawn great of interest for the exploration of extraordinary constitutive acoustic parameters [43–48]. The concept is first proposed by Liang and Li [43] and the corresponding design as a single curled unit is represented in Fig. 8a. Instead of using local resonance structures such as membranes or Helmholtz resonators which are suitable only for narrow frequency range devices, the authors achieved the negative refractive index over a broad range of frequency simply by coiling the space inside the metasurface and prism as can be seen in Fig. 8b. The structure consists of thin plates arranged in periodic channels. In Fig. 8a, the zigzag arrows on the left-hand side denote a path of waves in the second quadrant inside curled channels and X-shaped blue region on the right-hand side shows a simple view of the path of the waves through the curled channels. Through the dispersion relation derived by Floquet–Bloch theory, unusual properties such as negative, higher and zero refractive index could be indeed realized to satisfy the dispersion relation. Negative and higher index are obtained below the band-gap, whereas zero refractive index are obtained at nearly one point of frequency range which is exactly a band-gap frequency. In fact, each curled unit cell deliberately leads to propagate the air flow in curled channels and elongate the path of air flow. Therefore, the phase delay occurs along the elongated path, resulting in high refractive index. If a phase change is given with a negative value, then the negative refractive index can be obtained. Also, zero refractive index can be realized by squeezing waves inside the metasurface at a specific frequency, which shows a high transmission (Fig. 8c). This kind of symmetric geometry could be designed easily not only for two-dimensions but also for three-dimensions through the 3D printing technique.

### Acoustic cloaking

**Fig. 9:**
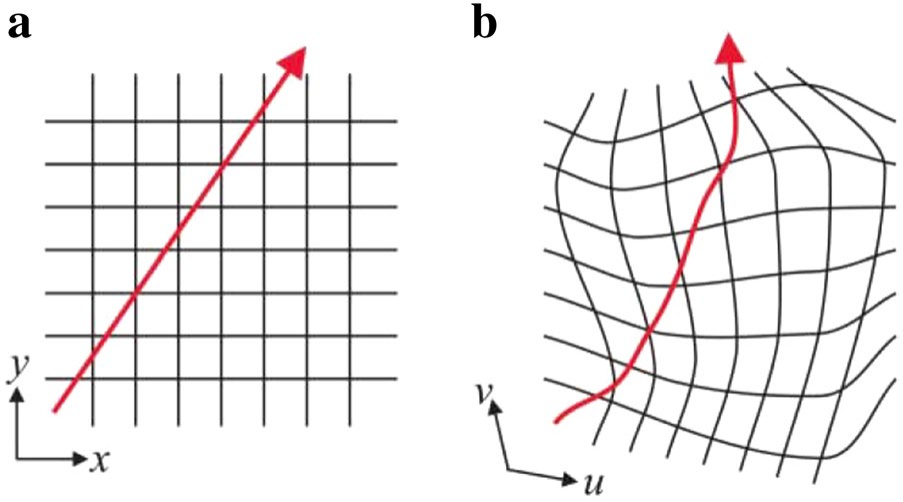
**a** A field line in free space with the background Cartesian coordinate grid shown. **b** The distorted field line with the background coordinates distorted in the same fashion. **a**, **b** are adapted from [58]

The advent of transformation optics has offered great versatility in designing acoustic metamaterials for deep manipulation of acoustic waves [56–64]. Not only macroscopic parameters such as transmission, absorption or reflection energy but also sub-wavelength spatial manipulation of the waves can be controlled. The idea is based on coordinate invariance of Maxwell’s equations on which the space of light can be squeezed and stretched by producing a desired spatial and right distribution of the permittivity and permeability through conformal transformations [58]. The metamaterial structures are designed thanks to the powerful ability of transformation optics to establish relationships between seemingly unrelated structures, particularly between complicated and simpler structures. For example, periodic plasmonic gratings can be generated from a simple slab through two conformal transformations [65], or high Q-factor whispering gallery modes are designed via transformation optics by linking to the fundamental whispery structure [66]. Figure 9 presents an example of the space distortion in the (*x*, *y*) plane of the Cartesian coordinates generated by conformal mapping.

By emerging this powerful tool into the acoustic wave science [67–69], acoustic applications for cloaking and super-resolution that require metamaterials containing complicated and hard to implement properties are now possible. The term “acoustic cloaking” refers to a phenomenon that a shell makes the surrounded object invisible from any directions of the incoming sound waves. In fact, the idea of acoustic cloaking was inspired from electromagnetics and optics where experimental cloaking phenomena have been realized at radio [59, 70] and optical [71] frequency range.

**Fig. 10:**
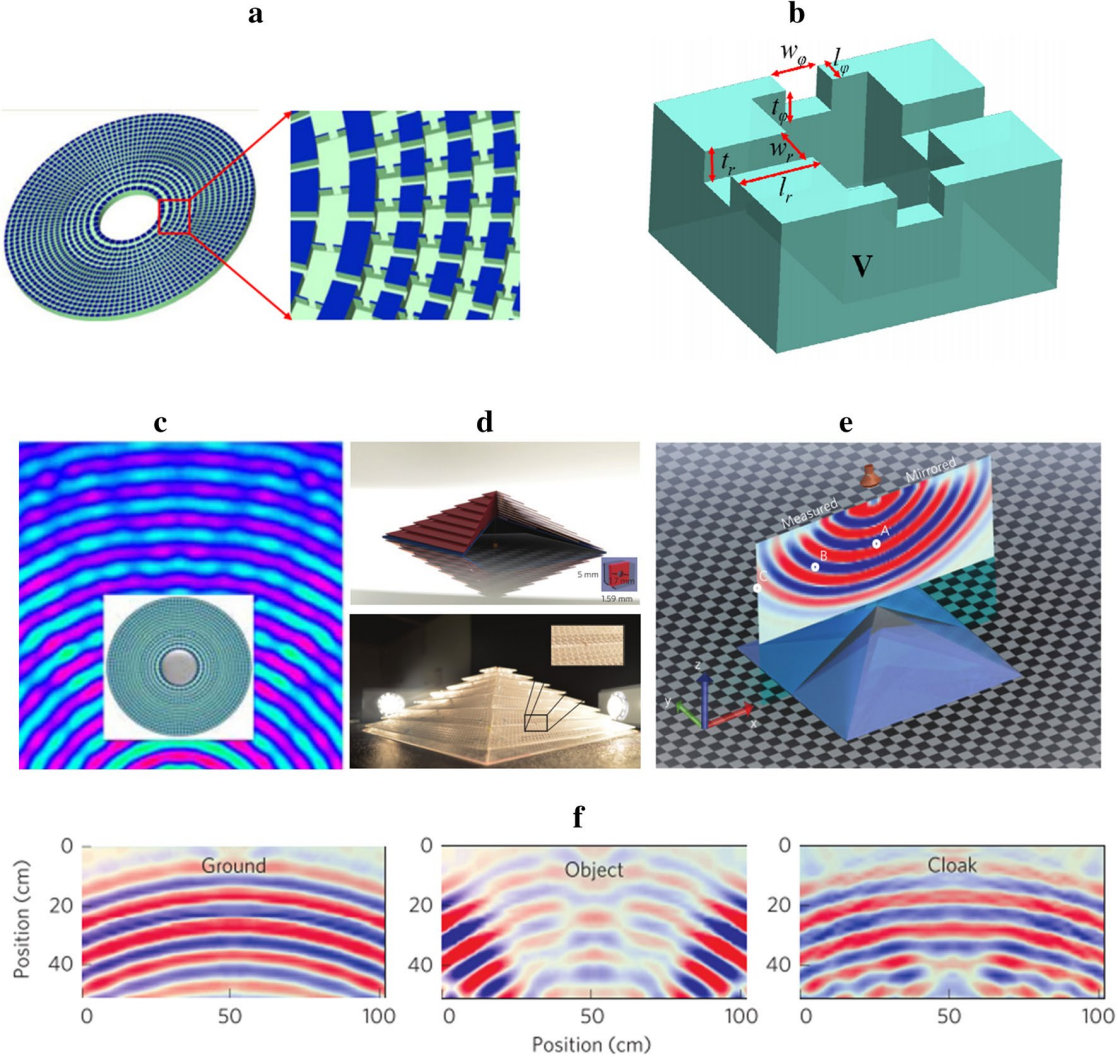
**a** The design of acoustic cloaking by Zhang et al. [16]. It consists of concentric layers with proper cavities and channels. **b** A unit cell of acoustic cloaking structure. The unit cell consists of a cavity with a large volume and four narrow channels as like as a shunt capacitor and serial inductors. **c** A measured pressure field plot at 64 kHz. The acoustic cloaking structure lies in the center of the water tank and the steel cylinder is positioned inside the structure. There is almost no scattering behind the structure, so that it shows the steel cylinder is well cloaked. **d** Scheme of physical structure designed (*above*) and a photograph of actual pyramid-shaped structure with perforated plastic plates (*below*). **e** Scheme of an experimental setup. There is a scanning microphone and *A*, *B* and *C* are specific points to be measured. **f** Instantaneous scattered pressure fields in each case. The case “Cloak” is well matched with “Ground” compared to “Object.” By arranging the pyramid-shaped structure, the inner space of the structure is recognized as empty one. **a**–**c** are adapted from [16] and **d**–**f** are adapted from [75]

The harmonic acoustic wave equation without a wave source is defined as

9 $$\nabla \cdot (B\nabla P) - \overset{\lower0.5em\hbox{$\smash{\scriptscriptstyle\leftrightarrow}$}} {\rho } \frac{{\partial^{2} P}}{{\partial t^{2} }} = 0$$

where $$\overset{\lower0.5em\hbox{$\smash{\scriptscriptstyle\leftrightarrow}$}} {\rho }$$ represents the mass density tensor. Based on the invariance of the acoustic wave equation under coordinate transformations, the equation in the transformed space can be written as

10 $$\nabla^{{\prime }} \cdot \left( {B^{{\prime }} \nabla^{{\prime }} P^{{\prime }} } \right) - \overleftrightarrow {\rho^{{\prime }} }\frac{{\partial^{2} P^{{\prime }} }}{{\partial t^{2} }} = 0$$

where $$\, \overset{\lower0.5em\hbox{$\smash{\scriptscriptstyle\leftrightarrow}$}} {\rho }^{\prime } = A\overset{\lower0.5em\hbox{$\smash{\scriptscriptstyle\leftrightarrow}$}} {\rho } A^{T} /\det (A) \,$$ and $$B^{\prime } = B[\det (A)].$$ *A* is the Jacobian matrix of coordinate transformation and, *A* ^*T*^ and det(*A*) denote transpose and determinant of *A*, respectively. Consequently, we can map the normal Cartesian space to a distorted space for the purpose of bending of wave propagation trajectories, resulting in the cloaking phenomenon. More theoretical details are presented in the work of Milton et al. [72] which described how to apply the cloaking phenomenon in electromagnetic waves to other types of waves, especially in the acoustic waves. Numerical studies for acoustic cloaking in two dimensions [13, 14] and three dimensions [15] have also been conducted. The first experiment of acoustic cloaking was realized by Zhang et al. [16] with a design of 2D array of sub-wavelength cavities filling with water and connected channels with spatially tailored geometry (Fig. 10a). The design of cavities is referred to the concept of lumped acoustic elements which are analogous to electronic circuit elements (Fig. 10b). As a result, 2D acoustic cloaking with a proper array of the unit cells composed of cavities and connected channels was achieved with almost no scattering in front and rare of the steel cylinder as shown in Fig. 10c.

Another approach for acoustic cloaking inspired from carpet cloaking suggested by Li and Pendry in electromagnetic field [73]. With this concept, the first experimental 2D acoustic carpet cloaking was demonstrated by Popa et al. [74]. Subsequently, a 3D carpet cloaking which is an extension of the 2D one was demonstrated by Zigoneanu et al. [75]. The setups of 2D and 3D carpet cloaking are made of arrays of the perforated plastic plates with sub-wavelength holes that allow the penetration of airborne sounds. Metamaterials with highly anisotropic mass density are required for this approach so that it can uncover high-loss scattering on the perforated plastic plates. For example, a 3D omnidirectional acoustic carpet cloaking was designed with a pyramid-shaped structure (Fig. 10d). The scheme of experimental setup is illustrated in Fig. 10e and the results of instantaneous scattered pressure field are shown in Fig. 10f. Besides the cloaking devices based on transformation acoustics, acoustic cloaking can also be realized by using scattering cancellation method to eliminate the scattered acoustic field between background and system [76–82].

### Acoustic lenses

Concepts of optical or electromagnetic lenses can also be applied to acoustics. In this sub-section, we will review multiple designs of acoustic metamaterials for realization of acoustic lenses, including superlens and hyperlens for sub-diffraction imaging, Luneburg lens for focusing acoustic waves without aberration and Eaton lens for control and manipulation of acoustic waves with arbitrary refraction angles in spherical geometry.

#### Superlens and hyperlens

**Fig. 11:**
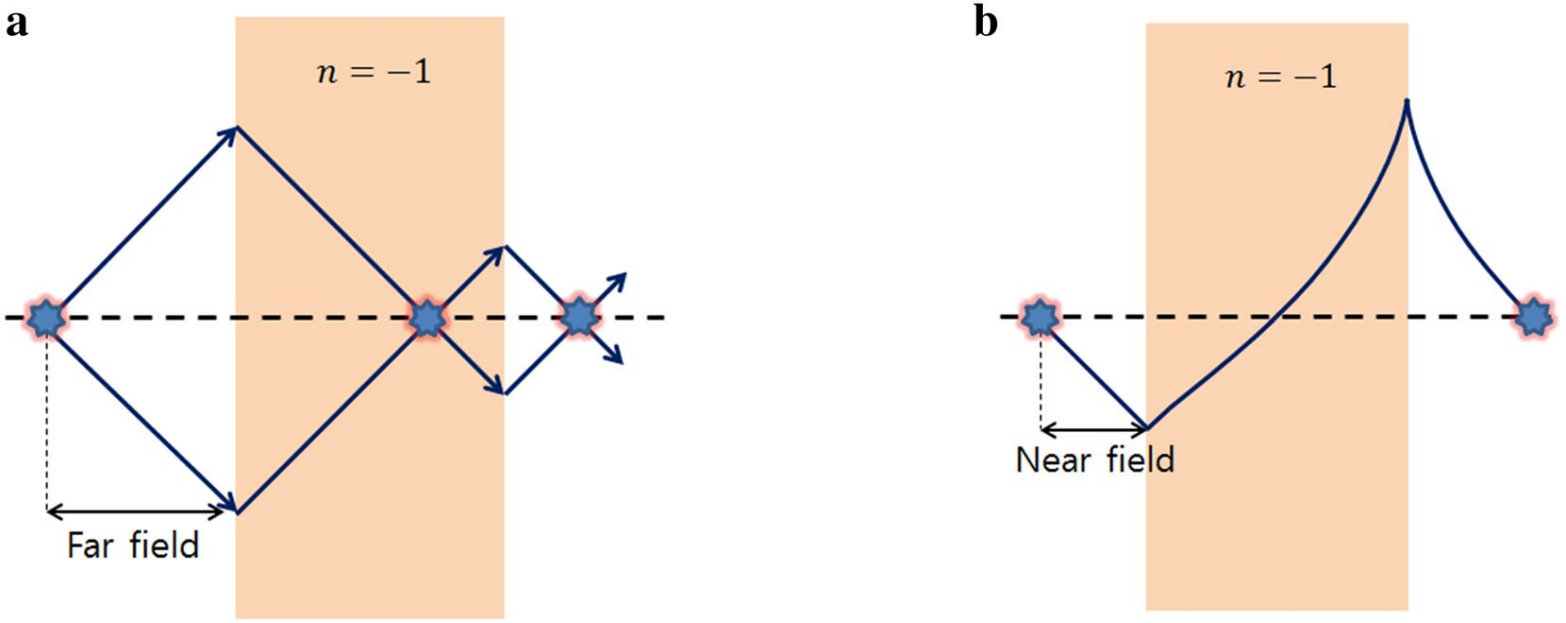
Scheme of wave manipulation by negative refractive index slab. **a** Diverging waves are focused in the far-field. **b** Enhancement of the wave amplitude in the near-field.

Superlens, hyperlens or, more completely, super-resolution lenses are devices which are able to image beyond the diffraction limit in both near- and far-field. In general, superlens and hyperlens are for the near-field and far-field, respectively. The concept of superlens was first proposed and demonstrated by Veselago–Pendry with a negative refractive index [1, 83] and has been the subject of intensive research due to a wide variety of applications in biology, pathology, medical science and nanotechnology. In his work, Pendry has shown that a negative index medium of superlens cannot converge diverging waves to a focal point in the far-field but can enhance their amplitude in the near-field (Fig. 11).

**Fig. 12:**
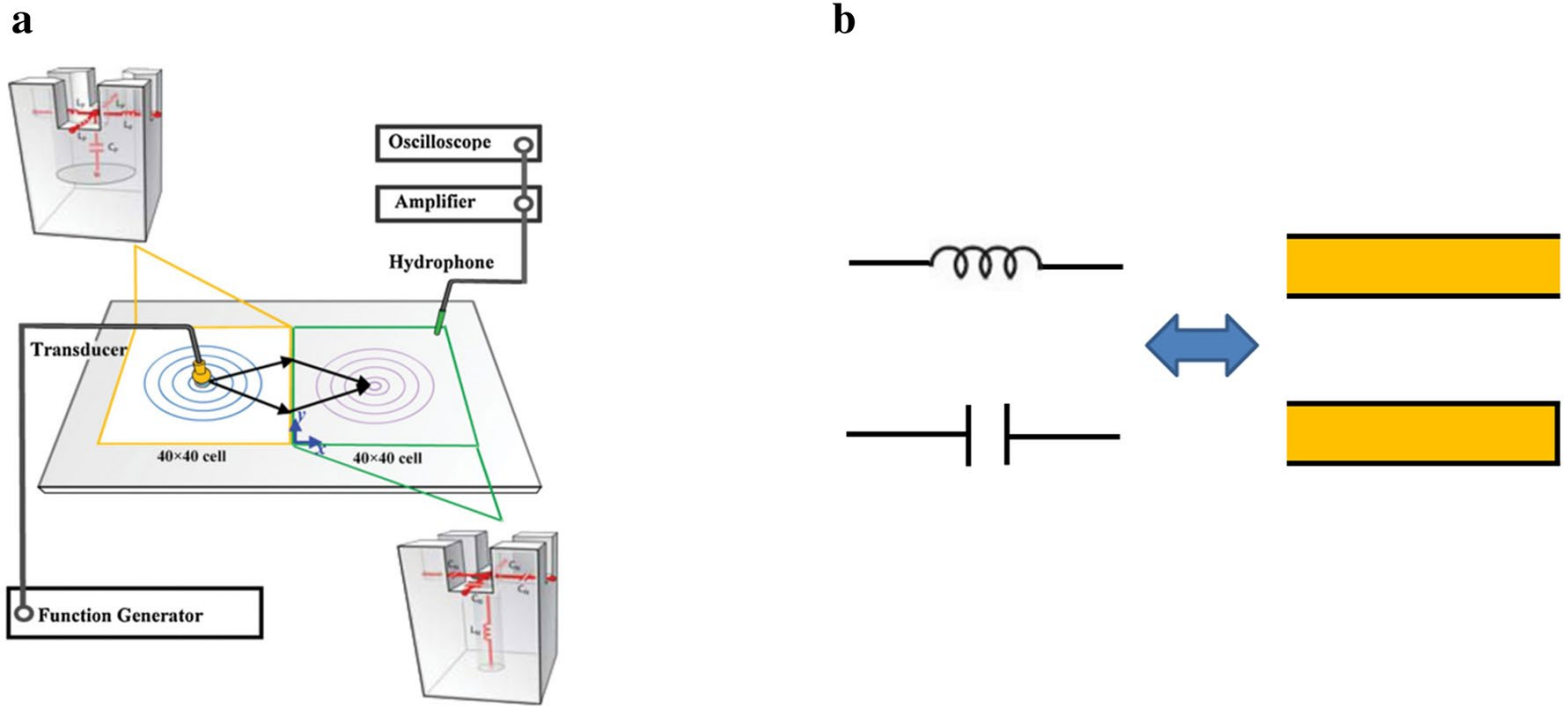
**a** An experimental setup for ultrasound focusing with PI–NI interface. **b** The inductor and capacitor are analogous to the open end and rigid end pipe, respectively. **a** is adapted from [84]

Figure 12a represents an experimental setup of the first demonstration of acoustic superlens with a negative refractive index [84]. This idea was based on Helmholtz resonators and originated from 2D transmission line method in electromagnetic metamaterials [85–87] by relating the effective mass density and bulk modulus of the network structure in the acoustic lumped circuit to the inductor and capacitor in the L-C circuit. The acoustic inductor (neck part) and capacitor (cavity part) are simply assumed to be an open end and rigid end pipe (Fig. 12b). By alternatively positioning acoustic inductors and capacitors, the negative refractive index can be acheived. Finally, perfect lens phenomenon which is understood as the focusing of ultrasound was realized by structuring different designed Helmholtz resonators for forming a PI-NI (positive index-negative index) interface.

Inherited from studies in optics [88–90], hyperlens which is known as artificial metamaterials with hyperbolic dispersion has been also applied to acoustics as an alternative way to overcome the diffraction limit of a given imaging system in the far-field regime. The principle of the acoustic hyperlens can be explained through the dispersion relation in acoustics as following

11 $$\frac{{k_{r}^{2} }}{{\rho_{\theta } }} + \frac{{k_{\theta }^{2} }}{{\rho_{r} }} = \frac{{\omega^{2} }}{B}$$

where *k*_*r*_, $k_{\theta }$ are wavevectors in the radial and azimuthal direction, respectively. In conventional medium, since both radial and tangential mass density are positive, the dispersion profile representing *k*_*r*_ as a function of $k_{\theta }$ will be circular according to Eq. (11) leading to the existence of a cutoff wavevector that limits the tangential spatial frequency, resulting in the diffraction limit. In the case of hyperlens, since $\rho_{r}$ is negative, the dispersion described in Eq. (11) will have a hyperbolic form in which the radial wavevector *k*_*r*_ can still be positive for a very large value of the tangential wavevector $k_{\theta } .$ In other words, the high frequency information of objects which cannot be resolved in the conventional system is transformed to propagating waves and brought to the far-field. Consequently, a magnified fine feature information can be acquired by using the hyperlens.

**Fig. 13:**
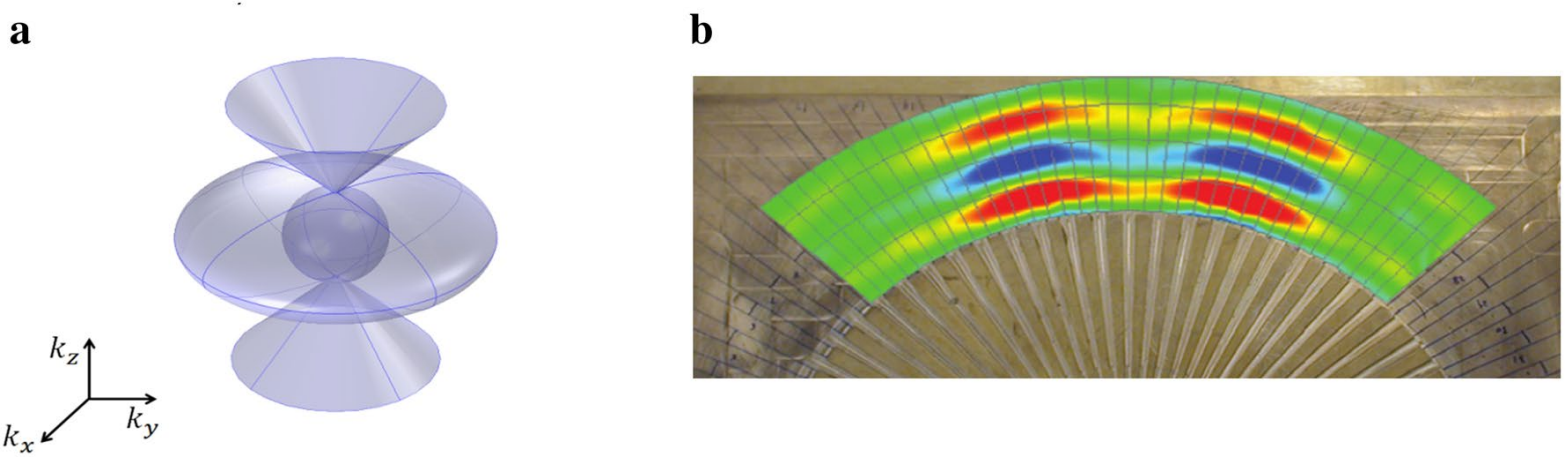
**a** *3D hyperbolic*, *elliptic* and *circle* isofrequency *contours*. The *circle contour* denotes a homogeneous material. The *elliptic* and *hyperbolic contours* are unusual cases which are not limited to the *circle* one. For *elliptic* case, there is a huge difference between the positive components. For hyperbolic case, there is a single negative component. **b** An experimental pressure measurement of an acoustic hyperlens. **b** is adapted from [9]

Li et al. [9] first demonstrated an acoustic hyperlens which is able to work for the broadband wave frequency with low loss. The hyperlens consists of alternating brass and air stripes along the *θ* direction (Fig. 13a). Because of the huge difference of mass densities between brass and air, highly anisotropic dispersion relation is obtained, leading to imaging enhancement as shown in Fig. 13b. The negative refractive index and enhanced imaging were also achieved by arranging proper layers of perforated plates with hyperbolic dispersion [10, 11]. More recently, Shen et al. [12] realized a hyperlens utilizing multiple arrays of clamped thin plates similar to membranes with the negative mass density, yielding a hyperbolic dispersion.

#### Luneburg and Eaton lens

**Fig. 14:**
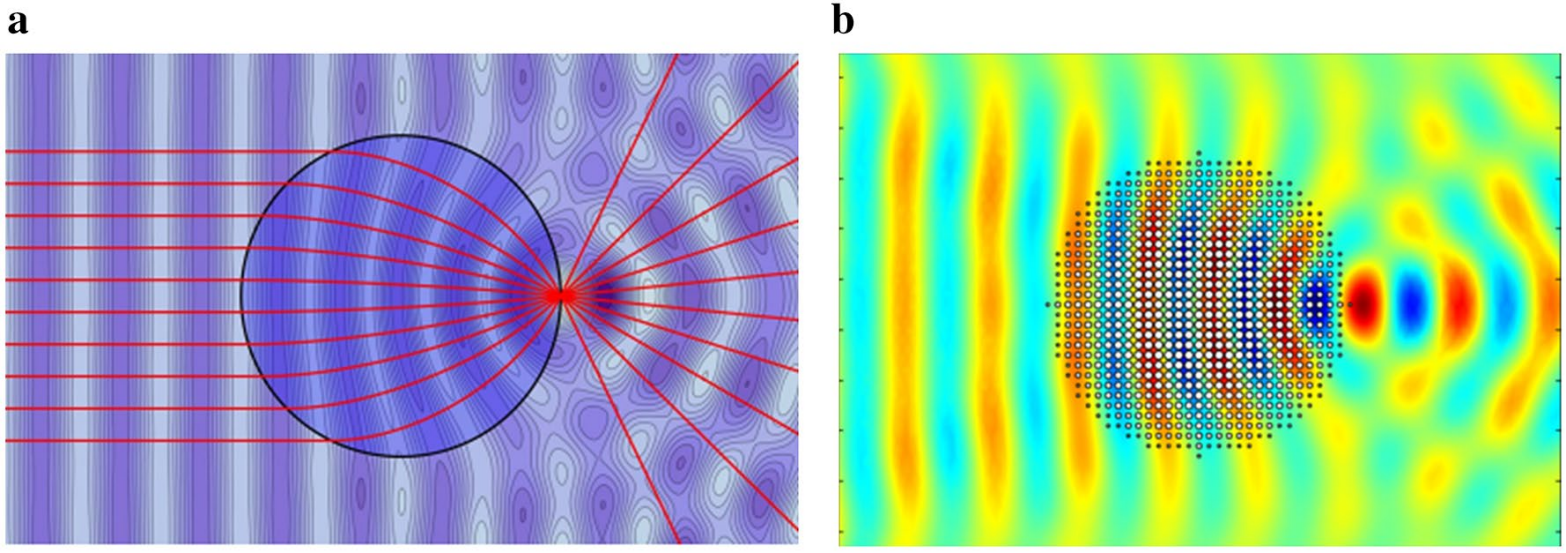
**a** An ideal Luneburg lens. **b** A designed acoustic Luneburg lens. Incoming sound waves from *left*-hand side focus on the opposite of the lens at 2000 Hz. **a** is adapted from [93], **b** is adapted from [97]

Luneburg lens is based on the concept of gradient index (GRIN) lens, in which refractive index decreases radially from the center to the outer surface [91–96]. For certain index profiles, the lens will form perfect geometrical images of two given concentric spheres onto each other, and are possible to guide and manipulate the incoming waves without aberration (see Fig. 14). In an ideal Luneburg lens (Fig. 14a), light trajectory (red rays) from various different positions can perfectly focus at one point without aberration.

Luneburg proposed this concept for the first time in 1940s [91], and it was well studied by Gutman [98], Morgan [99] in 1950s and Boyles [100] in 1960s. Zentgraf et al. [101] have realized the Luneburg lens in plasmonics. In acoustics, sound focusing based on GRIN lens was reported [102, 103] and diverse GRIN lenses for flexural waves were also demonstrated numerically [104]. First two-dimensional acoustic Luneburg lens has been reported by Kim [97, 105]. Such a Luneburg lens satisfies the equation of the refractive index given by a function of the radius

12 $$n(r) = \sqrt {2 - \left( {\frac{r}{R}} \right)^{2} }$$

where *R* is the radius of the lens and $0 \le r \le R.$ The wave equation of acoustic Luneburg lens is governed by mass density and bulk modulus. But, the bulk modulus inside and outside of the lens is assumed to be constant. Therefore, variable mass density inside of the lens is the main factor for acoustic Luneburg lens which can control the refractive index gradually. Recently, three-dimensional Luneburg lens was demonstrated at optical frequency range [106]. This kind of lenses in acoustics could be considered as a candidate for harvesting energy or sonar system in practical use.

Eaton lens as an extension of GRIN lens for arbitrary refraction angles in spherical geometry can also be realized  in acoustics by controlling the mass density inside of the lens with constant bulk modulus. 180° acoustic Eaton lens has been recently reported but, the complete demonstration still seems to be remained [107]. More efforts of metamaterial engineering are necessary for realization of Eaton lens which is able to work with various refraction angles.

## Conclusion

Together with the advent of electromagnetic and optical metamaterials, the field of acoustic metamaterials has expanded marvelously over the past 15 years. Although theoretical studies including analytical models and numerical tools have been well explored, many of significant challenges remain in the practical implementation of acoustic metamaterials. With the purpose to have a unified overview of the study progress, we have described research highlights with particular attention given to the sound waves in this review. Acoustic parameters, the mass density and bulk modulus, which are analogous to the permittivity and permeability in electromagnetic waves are identified as key parameters for acoustic wave science. We now know that various values of effective mass density and bulk modulus including negative values can be achieved by engineering mass-spring systems (or membranes) and Helmholtz resonators, respectively. Implementation of these structures for metamaterials with a single negative parameter, double negative parameters, near-zero and approaching-infinity mass density were then reviewed. In addition, space-coiling metamaterials were presented to realize negative, higher and zero refrative index not utilizing local resonance systems. We also reviewed some applications of acoustic cloaking with different approaches such as transformation acoustics, highly anisotropic parameters and scattering cancellation method. And then, superlens and hyperlens for diffraction limit breaking were well explained. Lastly, Luneburg and Eaton lens based on gradient index profile for manipulation of sound waves were introduced in terms of focusing and arbitrary refraction angles, respectively. Nowadays, acoustic metamaterials inspired by electromagnetic and optical metamaterials recently started influencing to not only elasticity but also seismology and even thermodynamics. Although our review didn’t include other fields of metamaterials, we also hope all research area of metamaterials will lead to advanced science and technology.
